# Investigating the magnetic, thermoelectric, and thermodynamic properties of the GeCH_3_ single-layer considering external magnetic field, doping, and strain

**DOI:** 10.1038/s41598-023-28430-5

**Published:** 2023-01-28

**Authors:** Mona Abdi, Bandar Astinchap

**Affiliations:** 1 Department of Physics, Faculty of Science, University of Kurdistan, Sanandaj, Kurdistan 66177-15175 Iran; 2 Research Center for Nanotechnology, University of Kurdistan, Sanandaj, Kurdistan 66177-15175 Iran

**Keywords:** Nanoscience and technology, Physics

## Abstract

Extensive research is ongoing to improve the performance of thermoelectric and thermodynamic properties of the material because preventing energy waste is vital in modern society. Herein, we study the thermoelectric and thermodynamic properties of the GeCH_3_ single-layer (SL) under the influence of an external magnetic field, electron doping, and tensile and compressive biaxial strain by using the tight-binding and equilibrium Green’s function method. We found that the electronic heat capacity, magnetic susceptibility, and electronic thermal and electrical conductivity increase by employing an external magnetic field, electron doping, and tensile biaxial strain. However, compressive biaxial strain yields a decrease in thermoelectric and thermodynamic properties. The results of our study show that the GeCH_3_ SL is paramagnetic. The results presented here that the GeCH_3_ SL is a suitable alternative for use in thermoelectric, spintronic, and valleytronics devices.

## Introduction

In the past decades, graphene has generated enthusiasm for research on two-dimensional (2D) materials^1–3^. One of the interesting characteristics of graphene is having high carrier mobility^4^, which is employed for electronic instruments. In addition, the zero band gap of graphene has yielded a series of restrictions in the application of microelectronic devices^5,6^. Therefore, the researcher's attempt was made to open the band gap of graphene and search for other 2D materials with appropriate carrier mobility and band gap. However, 2D materials such as transition-metal chalcogenides (MoS_2_, WS_2_… et.)^7,8^, Silicene^9^, Germanene^10^, and phosphorene^11,12^ have been studied by researchers. Among these 2D materials, MoS_2_ has a favorable band gap, but its carrier's mobility is low^13^. The phosphorene has a sufficient band gap, while the carrier mobility is poorly unstable^14^. Silicene and Germanene have attracted lots of study interest due to their high carrier mobility, low effective mass, and quantum spin Hall Effect^15–17^. However, the low band gap of Silicene and Germanene has restricted their use in electronic applications^18^. Recently, the GeH monolayer has been experimentally synthesized on SiO_2_/Si surface by mechanical exfoliation technique^19^. The GeH has enticed attention in the fields of optoelectronics and electronics due to having a band gap of 1.52 eV and low effective mass^20^. One of the disadvantages of the GeH is its low thermal stability, which leads to other surface terminations of the Germanene coming to scholars' attention^21^. Recently experimental studies on conductivity of the synthesized GeH monolayer show that GeH is a material with high mobility and low conductivity^22,23^. To solve this problem, a new 2D material GeH has recently been synthesized by substituting –H termination in GeH with –CH_3_ a material called the GeCH_3_. GeCH_3_ has an indirect gap of 1.69 eV, thermal stability to the temperature of 250 °C, and strong photoluminescence emission^24^. Liu et al. synthesized GeCH_3_ using a solvothermal method to investigate photocatalytic properties^25^. They demonstrated that GeCH_3_ has more suitable photocatalytic properties than GeH due to its lower charge transfer resistance, higher surface area, and preferable electronic structure. Livache et al. studied the optoelectronic properties of GeCH_3_ synthesized by the one-step covalent methyl-termination method^26^. Also, a series of studies have been performed on the electronic and structural properties of GeCH_3_ monolayer under strain effects^27,28^. Sheng et al. examined GeCH_3_ nanoribbons under strain effect via density functional theory (DFT)^29^. Their results exhibited that the band gap alters strongly with the amount of strain but does not change with the different widths. Due to the unique properties of GeCH_3_, it can be introduced as an important candidate for thermoelectric properties in the next generations. However, the thermodynamic and thermoelectric properties of the GeCH_3_ have not been reported. In this work, we study effects of the external magnetic field, biaxial strain, and electron doping on thermodynamic and thermoelectric properties of GeCH_3_SL in TB approximation. We used the Green function for calculating the electronic heat capacity, magnetic susceptibility, thermal conductivity, and electrical conductivity GeCH_3_SL. The frame of the remains of the paper is as follows. In section II, we report the TB model and Green’s function for calculating the total electronic heat capacity, magnetic susceptibility, thermal conductivity, and electrical conductivity. We discuss the numerical results related to the impacts of external magnetic field, biaxial strain, temperature, and electron doping on thermodynamic and thermoelectric properties in section III, and we pull the original results in section VI.

## Theory and model

**Figure 1:**
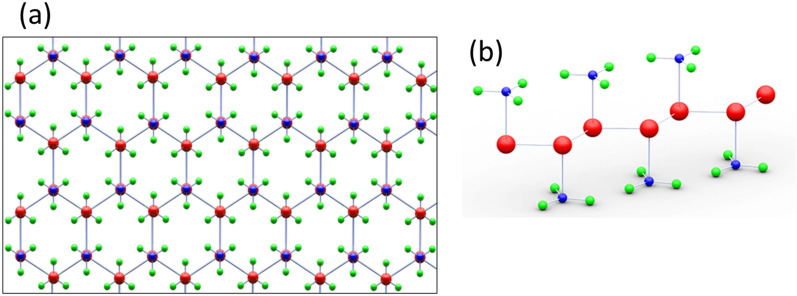
Graphic of (**a**) top and (**b**) side views of the GeCH_3_SL structure atomic.

The TB method is used as a functional strategy to study the properties of semiconductors. The TB method is a precise technique to determine the nature of bonds and the source of forces. The Hamiltonian of the TB model is calculated by the Slater-Koster (SK) parameters^30^. The SK parameters are utilized to compute the interaction between orbitals on neighboring atoms^31^. In the following, we study the thermoelectric and thermodynamic properties of the GeCH_3_SL via the TB model. According to Fig. 1, the GeCH_3_SL has a hexagonal structure, and its geometrical parameters have been determined. In Fig. 1b, we can see that the bulk hexagonal structure has three atomic layers and the Ge atom is sandwiched between the outer two methyl layers.

Based on DFT calculations, the GeCH_3_SL has a lattice constant of a = 3.9 A°, and the bond length between Ge–Ge and Ge-C atoms is 2.415 A° and 1.972 A°, respectively^28^. Also, the bulk height h between two various Ge sub-lattices is 0.788 A°. The DFT calculations exhibit that the electronic properties of the GeCH_3_SL are determined by s, p_x_, and p_y_ orbitals^27^. The effective Hamiltonian of the TB model in the nearest-neighbor approximation with the presence of the spin–orbit coupling and the external magnetic field is as follows:

1 $$\begin{aligned} H_{t.b}^{\sigma } &= \mathop \sum \limits_{i.j.\sigma .\alpha .\beta } \varepsilon_{i.\alpha }^{\sigma } \delta_{i.j} \delta_{\alpha \beta } a_{i.\alpha }^{\dag .\sigma } a_{j.\beta }^{\sigma } + \mathop \sum \limits_{i.j.\sigma .\alpha .\beta } t_{i.\alpha .j.\beta } a_{i.\alpha }^{\dag .\sigma } a_{j.\beta }^{\sigma } \hfill \\ &\quad- \mathop \sum \limits_{i.j.\sigma .\alpha .\beta } (g\mu_{B} {\text{B}}\sigma + {\upmu }) \delta_{i.j} \delta_{\alpha .\beta } a_{i.\alpha }^{\dag .\sigma } a_{j.\beta }^{\sigma } + \lambda \mathop \sum \limits_{i.\xi .\nu .\gamma .\sigma .\mathop \sigma \limits } a_{{ip_{\xi } \sigma }}^{\dag } a_{{ip_{\nu } \mathop \sigma \limits }} \left( { - i\varepsilon_{\xi \nu \gamma } \sigma_{\sigma \mathop \sigma \limits^{\prime} }^{\gamma } } \right). \hfill \\ \end{aligned}$$

where, $\alpha$ and $\beta$ refer to s, p_x_, p_y_ orbitals, ${a}_{i.\alpha }^{\dag.\sigma }({a}_{i.\alpha }^{\sigma })$ is the creation (annihilation) operator for electrons of $\alpha$ orbitals in the i atom. $\varepsilon_{i.\alpha }^{\sigma }$ is on-site energy of the $\alpha$ orbitals in the i atom and ${t}_{i.\alpha .j.\beta }$ indicate hopping energy from the $\alpha$ orbital of the i atom to the $\beta$ orbital of the j atom. The SK parameters for GeCH_3_SL are given in Table 1. In the third term ${\mu }_{B}$ and g represents the Bohr magneton and gyromagnetic constants, respectively, and B is an external magnetic field. In the fourth term $\lambda$ is spin–orbit coupling strength that its value is 0.096 eV^28^, ${\varepsilon }_{\xi \nu \gamma }$ defines the Levi–Civita symbol, and ${\sigma }_{\sigma \mathop \sigma \limits^{\prime} }^{\gamma }$ implies Pauli matrices.

**Table 1:** The SK parameters for GeCH_3_SL in the unit eV^28^.

${v}_{ss\sigma }$	${v}_{sp\sigma }$	${v}_{pp\sigma }$	${v}_{pp\pi }$	${E}_{s}$	${E}_{p}$
− 2.20	2.65	2.85	− 0.85	− 5.01	2.1

Using the energy spectrum obtained from the TB model Hamiltonian matrices, we can write the Hamiltonian by^32^:

2 $$H=\sum_{k.\sigma .\eta }{E}_{\eta }^{\sigma }{a}_{\eta .k}^{\dag.\sigma }{a}_{\eta .k}^{\sigma }.$$

Based on Eq. (2), the electron Green function offered as:

3 $${\text{G}}_{{\upeta }}^{{\upsigma }} \left( {{\text{k}}.{\uptau }} \right) = - \left\langle {{\text{T}}_{{\uptau }} \left( {{\text{C}}_{{\upeta }}^{{\upsigma }} \left( {{\text{k}}.{\uptau }} \right){\text{C}}_{{\upeta }}^{{{\upsigma }.\dag }} \left( {{\text{k}}.0} \right)} \right)} \right\rangle ;$$

here, τ refers to imaginary time, and the Fourier transformations of the electronic Green's function are achieved by:

4 $${\mathrm{G}}_{\upeta }^{\upsigma }\left(\mathrm{k}.\mathrm{ i}{\upomega }_{\mathrm{n}}\right)={\int }_{0}^{\frac{1}{{\mathrm{k}}_{\mathrm{B}}\mathrm{T}}}{\mathrm{e}}^{\mathrm{i\omega }{\uptau }_{\mathrm{n}}}{\mathrm{G}}_{\upeta }^{\upsigma }\left(\mathrm{k}.\uptau \right)=\frac{1}{\mathrm{i}{\upomega }_{\mathrm{n}}-{\mathrm{E}}_{\mathrm{\alpha }}^{\upsigma }}.$$

Applying strain to a system causes changes in its electronic properties and also leads to alter bond lengths and bond angles. In the following, we will examine the change of parameters hopping energy of the GeCH_3_SL in the presence of the biaxial strain. There are several strategies to discover this altered energy hopping^33^. We employed Harrison's law to modify the SK parameters for s and p orbitals that are proportionate to the square inverse of the distance i.e. ${V}_{\alpha \beta \gamma }\approx \frac{1}{{r}^{2}}$, which $\alpha$ and $\beta$ are the types of orbitals and $\gamma$ is the type of overlap between orbitals ($\sigma .\pi )$^12^.

When the biaxial strain applied to the GeCH_3_SL does not change its structure hexagonal, only the initial lattice vectors $\overrightarrow{{r}_{0}}$ deformed to $\overrightarrow{r}$. We can write $\overrightarrow{r}=\left(x\widehat{i}+y\widehat{j}+z\widehat{k}\right)$ vectors in terms of $\overrightarrow{{r}_{0}}=\left({x}_{0}\widehat{i}+{y}_{0}\widehat{j}+{z}_{0}\widehat{k}\right)$ vectors as follows:

5 $$x=\left(1+{\varepsilon }_{x}\right){x}_{0};\,\,\, y=\left(1+{\varepsilon }_{y}\right){y}_{0};\,\,\, z={z}_{0}.$$

In Eq. (5) ${\varepsilon }_{x}$ and ${\varepsilon }_{y}$ refers to the direction of strain in x and y, respectively. For simplicity, we assume that ${\varepsilon }_{y}={\varepsilon }_{x}=\varepsilon$. In the linear deformation regime, the development of a modified bond vector $\overrightarrow{r}$ as a function of the $\varepsilon$ offers as follows:

6 $$r\approx \left(1+{\alpha }_{x}{\varepsilon }_{x}+{\alpha }_{y}{\varepsilon }_{y}\right){r}_{0}=\left[1+\left({\alpha }_{x}+{\alpha }_{y}\right)\varepsilon \right]{r}_{0}.$$

here, ${\alpha }_{x}=\frac{{{x}_{0}}^{2}}{{{r}_{0}}^{2}}$ and ${\alpha }_{y}=\frac{{{y}_{0}}^{2}}{{{r}_{0}}^{2}}$ are the coefficients related to the geometrical system of the GeCH_3_single-layer, that ${\alpha }_{x}+{\alpha }_{y}={(\mathrm{cos}{\varphi }_{0})}^{2}$, and ${\varphi }_{0}$ is the initial bulk angle. Using Harrison's law for SK parameters under biaxial strain written as follows:

7 $$\begin{aligned} V_{\alpha \beta \gamma } &= \frac{C}{{r_{0}^{2} \left( {1 + 2\varepsilon \left( {cos\varphi_{0} } \right)^{2} } \right)}} \approx \frac{C}{{r_{0}^{2} }}\left( {1 - 2\varepsilon \left( {cos\varphi_{0} } \right)^{2} } \right) \hfill \\& = V_{\alpha \beta \gamma }^{0} \left( {1 - 2\varepsilon \left( {cos\varphi_{0} } \right)^{2} } \right). \hfill \\ \end{aligned}$$

In Eq. (7), C is a multiplicative constant, and ${V}_{\alpha \beta \gamma }^{0}$ parameters of SK are not modified. So that Eq. (7) determines the modified SK parameters, according to which we can calculate the physical properties of strained GeCH_3_SL. In the following, we present the thermoelectric and thermodynamic properties of the GeCH_3_SL using electron Green's function^34^. To compute the electronic heat capacity and magnetic susceptibility, we first calculate the density of states (DOS), which is described by:

8 $${\text{DOS}}\left( {\text{E}} \right) = - \frac{1}{{2{\pi N}}}\mathop \sum \limits_{{{\text{k}}.{\text{n}}.{\upsigma }.{\upeta }}} {\text{Im}}\left( {{\text{G}}_{{\upeta }}^{{\upsigma }} \left( {{\text{k}}.{\text{i}\omega }_{{\text{n}}} \to {\text{E}} + {\text{i}}0^{ + } } \right)} \right).$$

By defining the DOS, we can compute the total magnetic susceptibility and electronic heat capacity^35–37^. However, the electronic heat capacity and magnetic susceptibility in terms of DOS are obtained as follows:

9 $$\begin{aligned} {\text{C}}\left( {\text{T}} \right) & = \mathop \smallint \limits_{ - \infty }^{ + \infty } {\text{dE}}\left( {\frac{{\partial {\text{n}}_{{\text{f}}} }}{{\partial {\text{T}}}}} \right){\text{E DOS}}\left( {\text{E}} \right). \hfill \\ {\upchi }\left( {\text{T}} \right) & = \mathop \smallint \limits_{ - \infty }^{ + \infty } {\text{dE}}\left( {\frac{{ - \partial {\text{n}}_{{\text{f}}} \left( {\text{E}} \right)}}{{\partial {\text{E}}}}} \right){\text{DOS}}\left( {\text{E}} \right). \hfill \\ \end{aligned}$$

In Eq. (9), ${\mathrm{n}}_{\mathrm{f}}=\frac{1}{1+{\mathrm{e}}^{\upbeta (\mathrm{E}-\upmu )}}$ is the Fermi–Dirac distribution function. Now we calculate thermal, Seebeck coefficient, and electrical conductivity utilizing transport coefficients, which we have^38,39^:

10 $$\begin{aligned} {\upkappa } &= \left( {\frac{1}{{T^{2} }}} \right){ }\left( {{\text{L}}_{22} - \frac{{({\text{L}}_{12}^{2} )}}{{{\text{L}}_{11} }}} \right). \hfill \\ {\text{S}} &= - \frac{1}{{\text{T}}}\left( {\frac{{{\text{L}}_{12} }}{{{\text{L}}_{11} }}} \right). \hfill \\ {\upsigma } &= e^{2} {\text{L}}_{11} . \hfill \\ \end{aligned}$$

That the transport coefficients according to the electron Green's function are defined as follows:

11 $$\begin{aligned} {\text{L}}_{11}& = \frac{{{\text{e}}^{2} }}{{2{\beta V}}}\mathop \sum \limits_{k.\eta .\sigma } \left(\frac{{\partial E_{\eta .\sigma } }}{{\partial k_{x} }}\right)^{2} \mathop \int \limits_{ - \infty }^{ + \infty } \frac{d\varepsilon }{{2{\uppi }}}\left( { - \frac{{\partial {\text{n}}_{{\text{f}}} \left( \varepsilon \right)}}{\partial \varepsilon}} \right)( - 2Im({\text{G}}_{\eta .\sigma } \left( {k.i\omega_{n} \to \varepsilon + i0^{ + } } \right)^{2} . \hfill \\ {\text{L}}_{12} &= \frac{{\text{e}}}{{2{\beta V}}}\mathop \sum \limits_{k.\eta .\sigma } \left(\frac{{\partial E_{\eta .\sigma } }}{{\partial k_{x} }}\right)^{2} \mathop \int \limits_{ - \infty }^{ + \infty } \frac{d\varepsilon}{{2{\uppi }}}\varepsilon \left( { - \frac{{\partial {\text{n}}_{{\text{f}}} \left(\varepsilon \right)}}{\partial \varepsilon}} \right)( - 2Im({\text{G}}_{\eta .\sigma } \left( {k.i\omega_{n} \to \varepsilon + i0^{ + } } \right)^{2} . \hfill \\ {\text{L}}_{22} &= \frac{1}{{2{\beta V}}}\mathop \sum \limits_{k.\eta .\sigma } \left(\frac{{\partial E_{\eta .\sigma } }}{{\partial k_{x} }}\right)^{2} \mathop \int \limits_{ - \infty }^{ + \infty } \frac{d\varepsilon }{{2{\uppi }}}\left( {\varepsilon^{2} } \right)\left( { - \frac{{\partial {\text{n}}_{{\text{f}}} \left( \varepsilon \right)}}{\partial \varepsilon }} \right)( - 2Im({\text{G}}_{{{\upeta }.{\upsigma }}} \left( {k.i\omega_{n} \to \varepsilon + i0^{ + } } \right)^{2} . \hfill \\ \end{aligned}$$

In the next section, we examine the numerical consequences of the thermoelectric and thermodynamic properties of the GeCH_3_SL in the presence of electron doping, strain, and external magnetic fields.

## Numerical results

**Figure 2:**
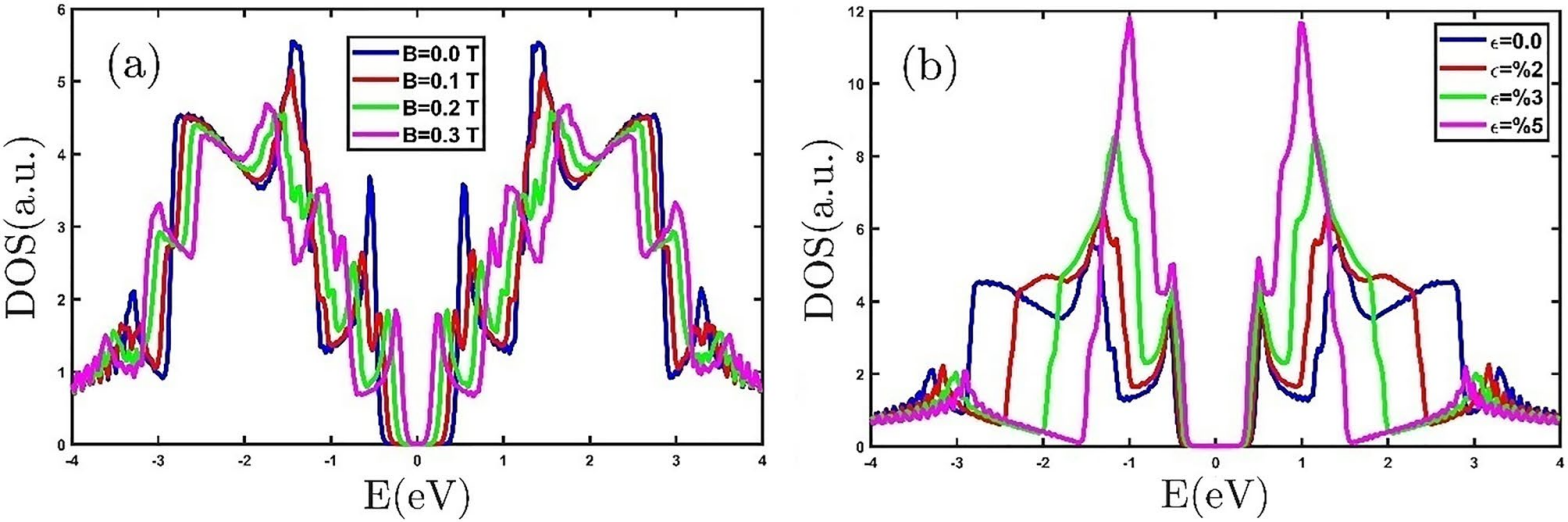
The electronic DOS of the GeCH_3_ SL (**a**) in the presence of different external magnetic fields and (**b**) for different tensile biaxial strains.

Due to its high thermal stability, the GeCH_3_ SL is used as a topological insulator candidate at high temperatures. The thermoelectric and thermodynamic properties of the GeCH_3_SL have not been examined yet. Therefore, we investigate the thermoelectric and thermodynamic properties of the GeCH_3_SL under the effect of the external magnetic field, electron doping, and biaxial strain by employing the TB model. The electronic DOS of the GeCH_3_ SL with different external magnetic fields is shown in Fig. 2a. We can see that GeCH_3_ SL has a band gap at E = 0 of the Fermi level. In Fig. 2a, the external magnetic field changes the number and position of the peaks. Due to the Zeeman effect, increasing the external magnetic field increases the split of the peaks, which reduces the band gap^37^. In other words, the Fermi energy approaches the van Hove energy by applying an external magnetic field, and the VHS peak in DOS splits into two new ones. Our results are consistent with the work done on the MoS_2_ monolayer and SiC bilayer^37,39^. The GeCH_3_ SL change from semiconductor to the conductor as the external magnetic field increases. Figure 2b shows the electronic DOS of GeCH_3_ SL in the presence of different tensile biaxial strains. As we can see, tensile biaxial strains reduce the band gap. These results have been confirmed by Yandong et al^27^.

**Figure 3:**
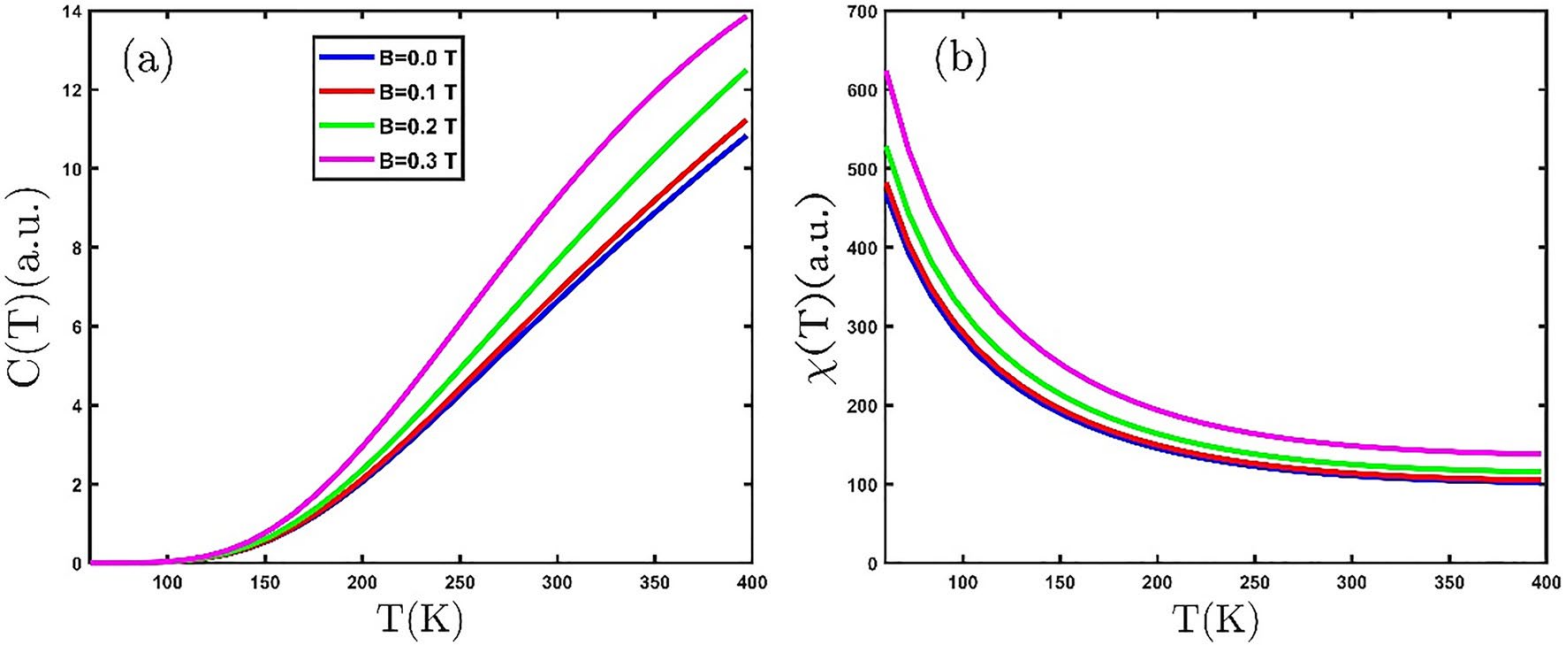
(**a**) The electronic heat capacity (**b**) magnetic susceptibility of the GeCH_3_SL versus temperature for various external magnetic fields.

In Fig. 3a, the electronic heat capacity for the GeCH_3_SL is illustrated as a function of temperature in the presence of various external magnetic fields. The electronic heat capacity increases by growing temperature in Fig. 3a because the kinetic energy of electrons rises with increasing temperature, which yields electrons to transfer to the conduction band. It is observed in Fig. 3a that at a constant temperature, the electronic heat capacity has risen with the increase of the external magnetic field. Because the external magnetic field, by applying force to the electrons, causes them to move from the valence band to the conduction band. These results are confirmed by Abdi et.al^8^. The magnetic properties of materials are determined via their magnetic susceptibility. Magnetic susceptibility is the ability of a material to be magnetized in a magnetic field. Materials are classified according to their magnetic susceptibility into diamagnetic ($\chi <0$), paramagnets, ferromagnetic, and antiferromagnetic ($\chi >0$). Figure 3b depicts variations in the magnetic susceptibility of the GeCH_3_SL with temperature for different values of the external magnetic field. According to the Curie–Weiss law for paramagnetic materials the magnetic susceptibility is $\chi \approx \frac{1}{T}$^40^. In Fig. 3b, we can see that the GeCH_3_SL is a paramagnetic material. As we know, an external magnetic field can order the magnetic dipoles; for this reason, the magnetic susceptibility has grown with the increase of the external magnetic field in Fig. 3b.

**Figure 4:**
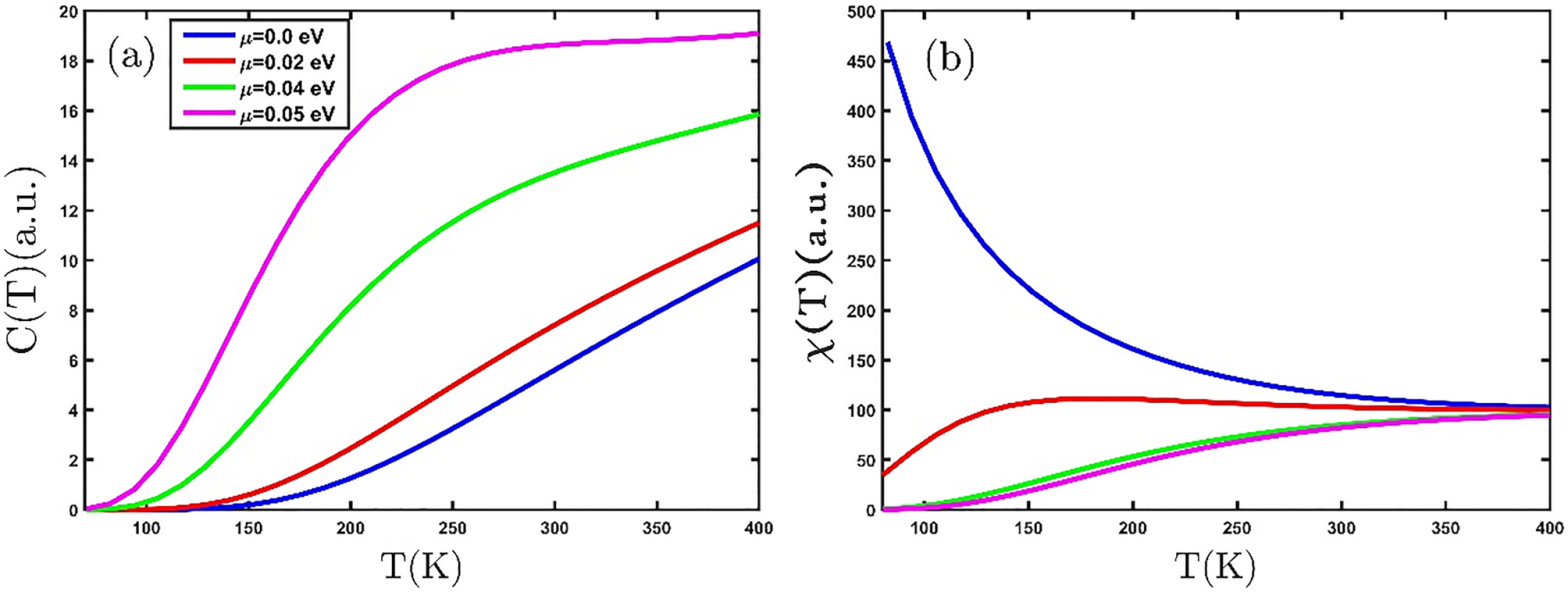
(**a**) The electronic heat capacity (**b**) magnetic susceptibility of the GeCH_3_SL versus temperature for various electron doping.

Now, we offer the effects of electron doping on the numerical results of the thermodynamic properties of the GeCH_3_SL. The dependence of the electronic heat capacity of the GeCH_3_SL on temperature under the influence of electron doping is displayed in Fig. 4a. As can be seen, the electronic heat capacity rises with the growing temperature. Rising temperature cause an increase in the electronic heat capacity, because of better electron repositioning from the valence band to the conduction band. Consequently, electron doping increases the population of electrons in the conduction band. Based on the curve's variation in Fig. 4a, it can conclude that electron doping increases the electronic heat capacity. In the electron doping value of $\mu =0.05 eV$, we can observe that the Schottky anomaly emerges in the electronic heat capacity. Figure 4b exhibits the magnetic susceptibility of the GeCH_3_SL in terms of temperature at different values of electron doping. According to the value of magnetic susceptibility in Fig. 4b, the GeCH_3_SL is a paramagnetic material. By applying electron doping, the electronic heat capacity curve changes with temperature. In the electron doping of $\mu =0.02 eV$, the magnetic phase of the material alters to antiferromagnetic, and its Neel temperature is 150 K. For fixed temperature, the magnetic susceptibility decreases with increasing electron doping in Fig. 4b; Because electron doping increases the population of electrons in the conduction band. As a result, the ordering of the dipoles decreases and leads to a lowering in magnetic susceptibility.

**Figure 5:**
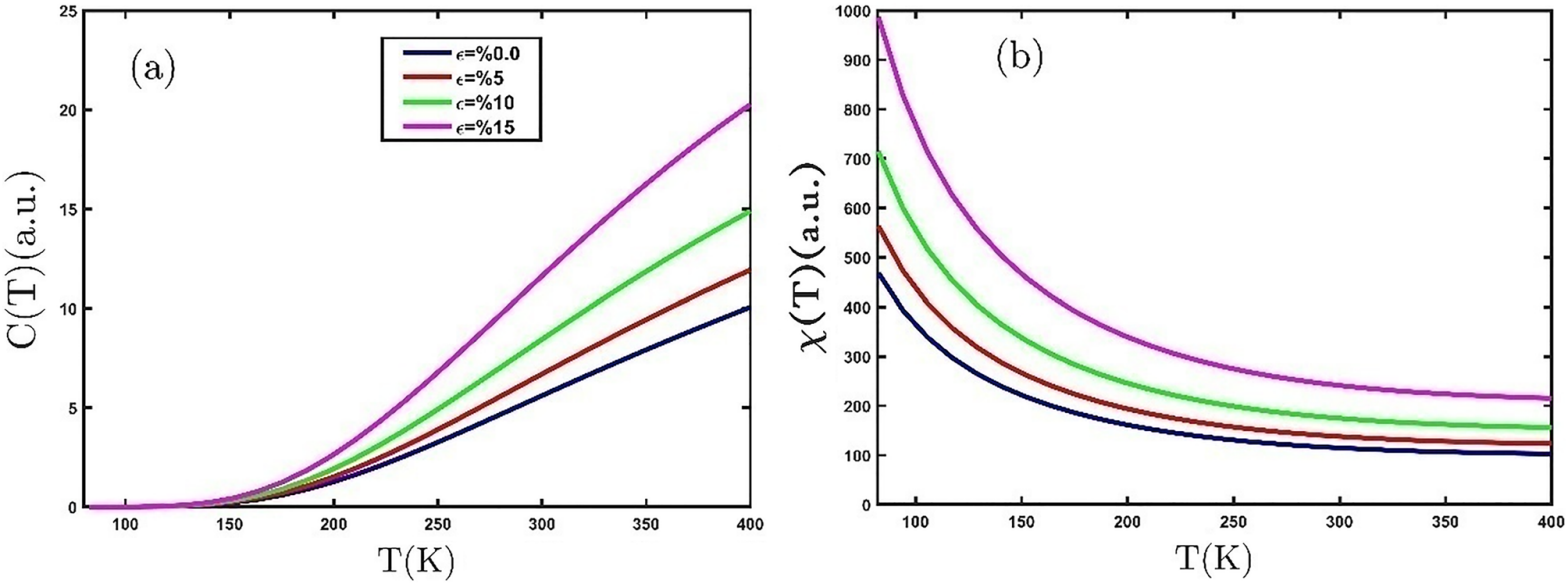
(**a**) The electronic heat capacity (**b**) magnetic susceptibility of the GeCH_3_SL as a function of the temperature for different tensile biaxial strains.

Strain yields modifications in the length and angle of bonds. Therefore, the strain alters the SK parameters, which leads to changing the properties of the material. In the following, we study the effects of tensile biaxial strain on the thermodynamic properties of the GeCH_3_SL. Figure 5a displays the temperature dependence of the electronic heat capacity of the GeCH_3_SL for different tensile biaxial strains. The behavior of electronic heat capacity rises with temperature. As we can observe in Fig. 5a, applying tensile biaxial strain increases the electronic heat capacity. The tensilebiaxial strain reduces the band gap in the GeCH_3_ SL^28^. According to Einstein-Debye law $C\left(T\right)\sim {e}^{\left(-\frac{{E}_{g}}{{k}_{B}T}\right)}$^35^, a drop in the band gap increases the electronic heat capacity. These results are confirmed by Liu et.al^41^. Figure 5b shows the magnetic susceptibility of the GeCH_3_SL vs. temperature for various tensilebiaxial strains. Based on the Curie–Weiss law, the GeCH_3_SL is a paramagnetic material, because the magnetic susceptibility reduces with temperature inverse. Increases magnetic susceptibility by applying tensilebiaxial strain at a constant temperature, exhibiting that tensilebiaxial strain generates the arrangement of magnetic dipoles at room temperature.

**Figure 6:**
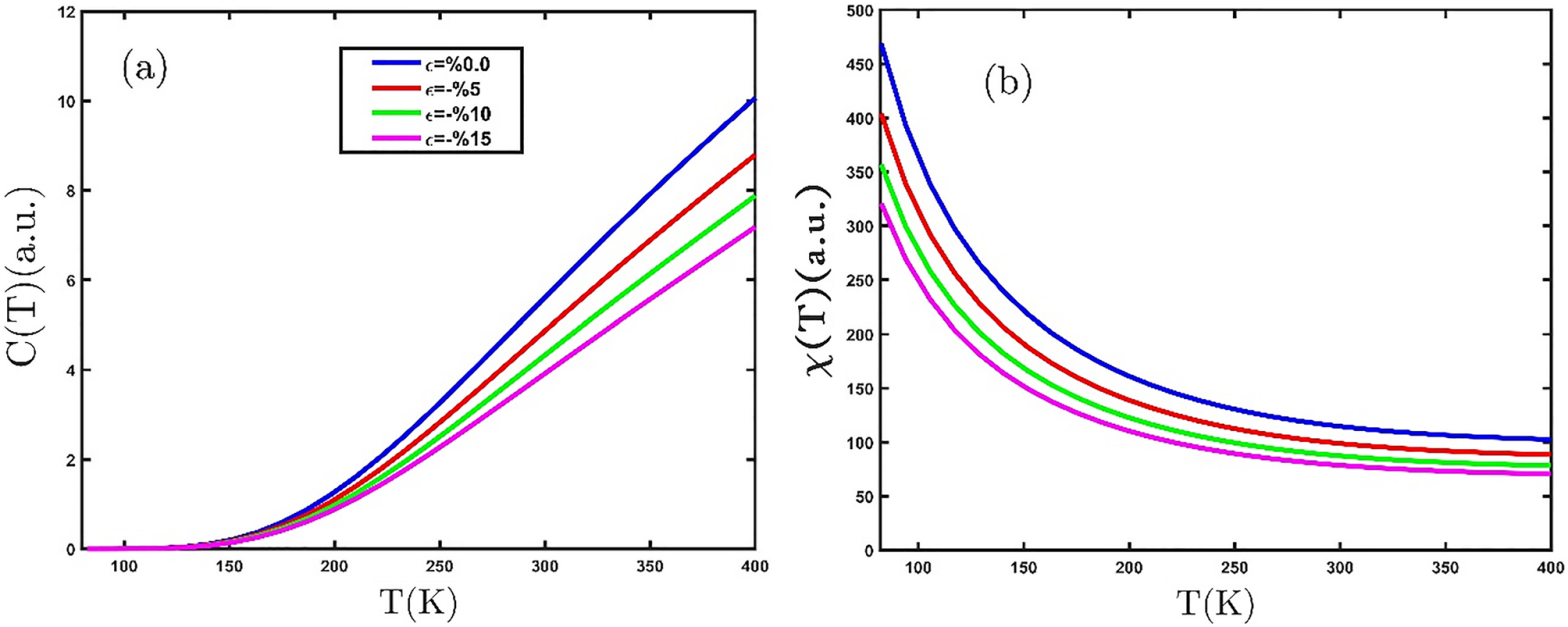
(**a**) The electronic heat capacity and (**b**) magnetic susceptibility of the GeCH_3_SL as a function of the temperature for different compressive biaxial strains.

We are going to investigate the effect of the compressive biaxial strain on the thermodynamic properties of the GeCH_3_SL. The electronic heat capacity is a vital parameter to consider the thermodynamic properties of materials. The electronic heat capacity as a function of temperature by applying compressive biaxial strain is plotted in Fig. 6a. The electronic capacity rises exponentially with an increase in temperature. We can find from Fig. 6a that the electronic heat capacity reduces with the increase of compressive biaxial strain. In Fig. 6b, the compressive biaxial strain is applied to the magnetic susceptibility. It can be observed from Fig. 6b that as the compressive biaxial strain grows; the magnetic susceptibility decreases. This shows that the compressive biaxial strain reduces the order and orientation of the dipoles.

**Figure 7:**
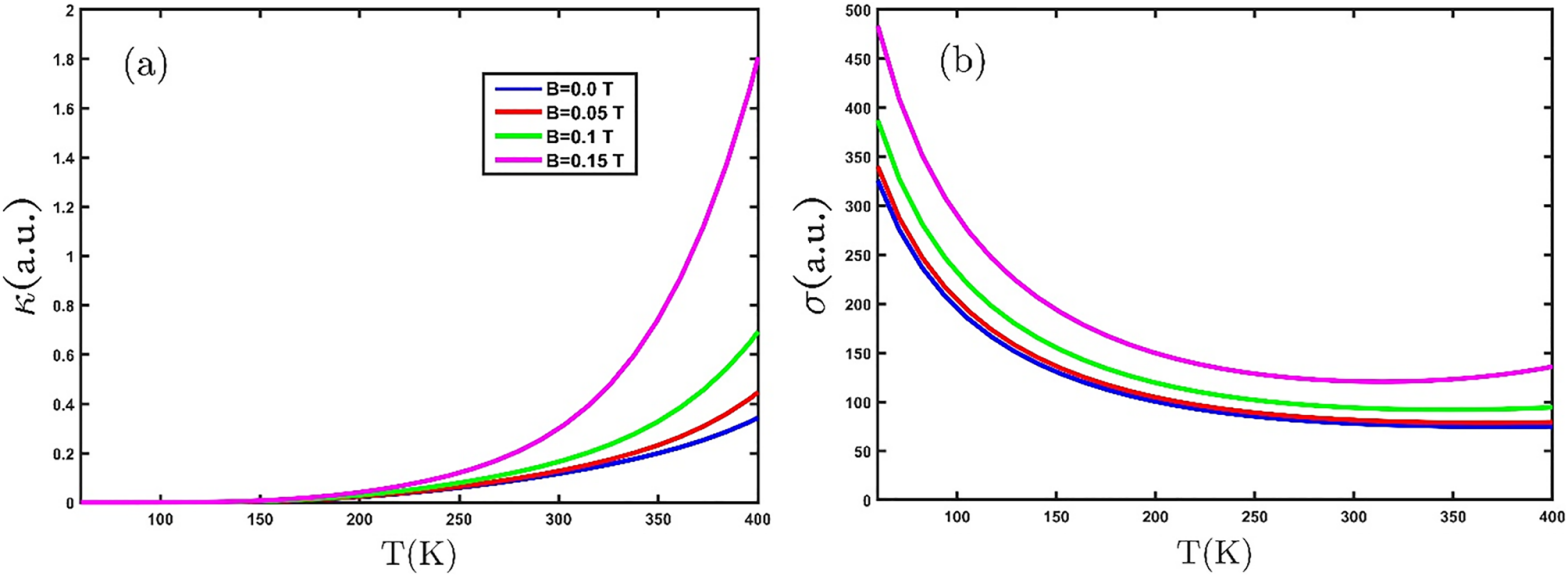
(**a**) and (**b**) The electronic thermal and electrical conductivity in terms of temperature under the effect of various external magnetic fields, respectively.

The electronic thermal conductivity of the GeCH_3_SL in the temperature range of 400 K under the effect of different external magnetic fields can be seen in Fig. 7a. At low temperatures, due to the lack of allowed charge carriers, there is not enough energy to transition charge carriers from the valance band to the conduction band, so the value of electronic thermal conductivity is zero. On the other hand, in the zero external magnetic fields, increasing the temperature increases the scattering rate of the carriers, as a result, the inter-band transition increases. Further, with applying of the stronger external magnetic field, the growth rate of electronic thermal conductivity has increased due to the increase in order and proper orientation of charge carriers. Figure 7b shows the electrical conductivity of the GeCH_3_SL in terms of temperature under the effect of different external magnetic fields. At T = 0 K, with an increase in the intensity of the external magnetic field, the dynamics of charged particles are limited along the direction of the magnetic field (magnetic energy is dominant), as a result of the inter-band transition of organized charge carriers, electrical conductivity increases. In the lower temperature range (lower than room temperature), the electrical conductivity decreases with increasing temperature.

**Figure 8:**
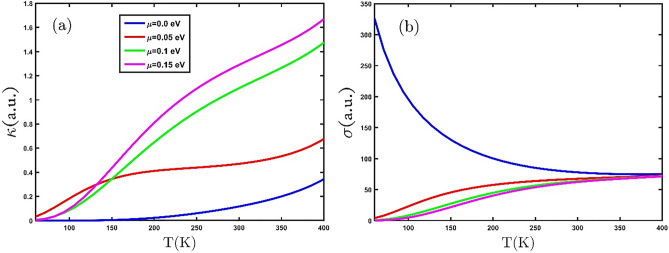
(**a**) The electronic thermal conductivity and (**b**) electrical conductivity of the GeCH_3_SL under the effect of electron doping in terms of temperature.

As the population of electrons increases, the rate of inter-band transitions will increase. But an increase in the population of electrons can increase (decrease) the affected quantity depending on the type of property being calculated. Figure 8a and b shows the electronic thermal conductivity and electrical conductivity of the GeCH_3_SL under the effect of electron doping in terms of temperature, respectively. It can be seen that the electronic thermal conductivity increases with increasing electron doping (Fig. 8a). This increase in electronic thermal conductivity is since the kinetic energy of the charge carriers increases with rising electron doping and as a result, inter-band transitions increase. Figure 8b shows the changes in electrical conductivity with temperature under various electron doping. It is seen that the electrical conductivity behavior at low and high temperatures is different in the presence and absence of electron doping. The results show that electrical conductivity is at its highest value at T = 0 and $\mu =0$. Moreover, with increasing electron doping, electrical conductivity decreases in the lower temperature range. At $\mu =0$, with the increase in temperature, the electrical conductivity decreases owing to the increase in the scattering rate of electrons and decrease in the transition energy level.

**Figure 9:**
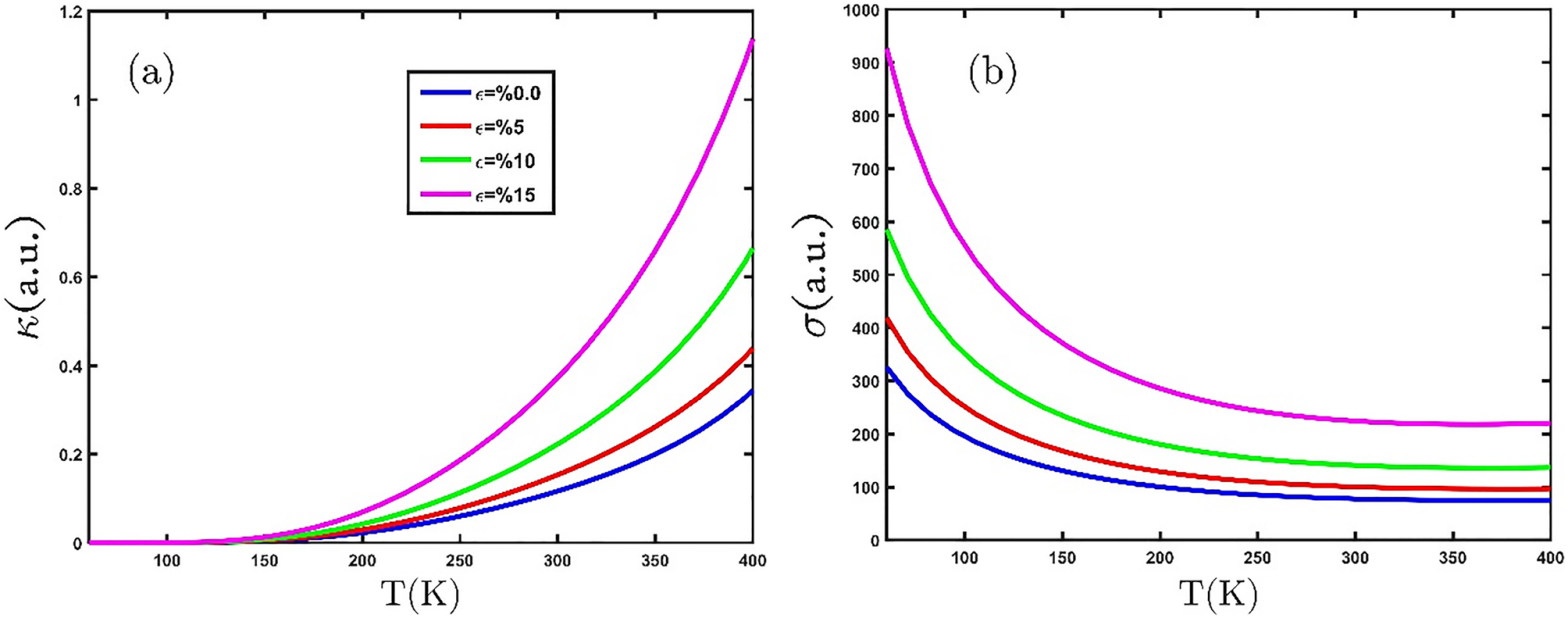
(**a**) and (**b**) The electronic thermal and electrical conductivity as a function of temperature under the effect of various tensile biaxial strains, respectively.

In the following, the result of the effect of the biaxial strain on the thermoelectric properties of the GeCH_3_SL is presented. Figure 9a exhibits the dependence of the electronic thermal conductivity on the temperature in the presence of various tensile biaxial strains. As we can see in Fig. 9a, the electronic thermal conductivity grows with increasing temperature. When the temperature increases, the kinetic energy of electrons raise and results in the electrons move to the conduction band. Furthermore, an increase in tensile biaxial strain yields increase in electronic thermal conductivity. This result shows that the tensile biaxial strain reduces the band gap and causes an increase in electronic thermal conductivity. The electrical conductivity of the GeCH_3_SL is plotted in Fig. 9b in terms of temperature by employing tensile biaxial strain. It can be seen that the electrical conductivity in the absence of strain decreases with increasing temperature. When tensile biaxial strain is applied, electrical conductivity rises at an invariant temperature.

**Figure 10:**
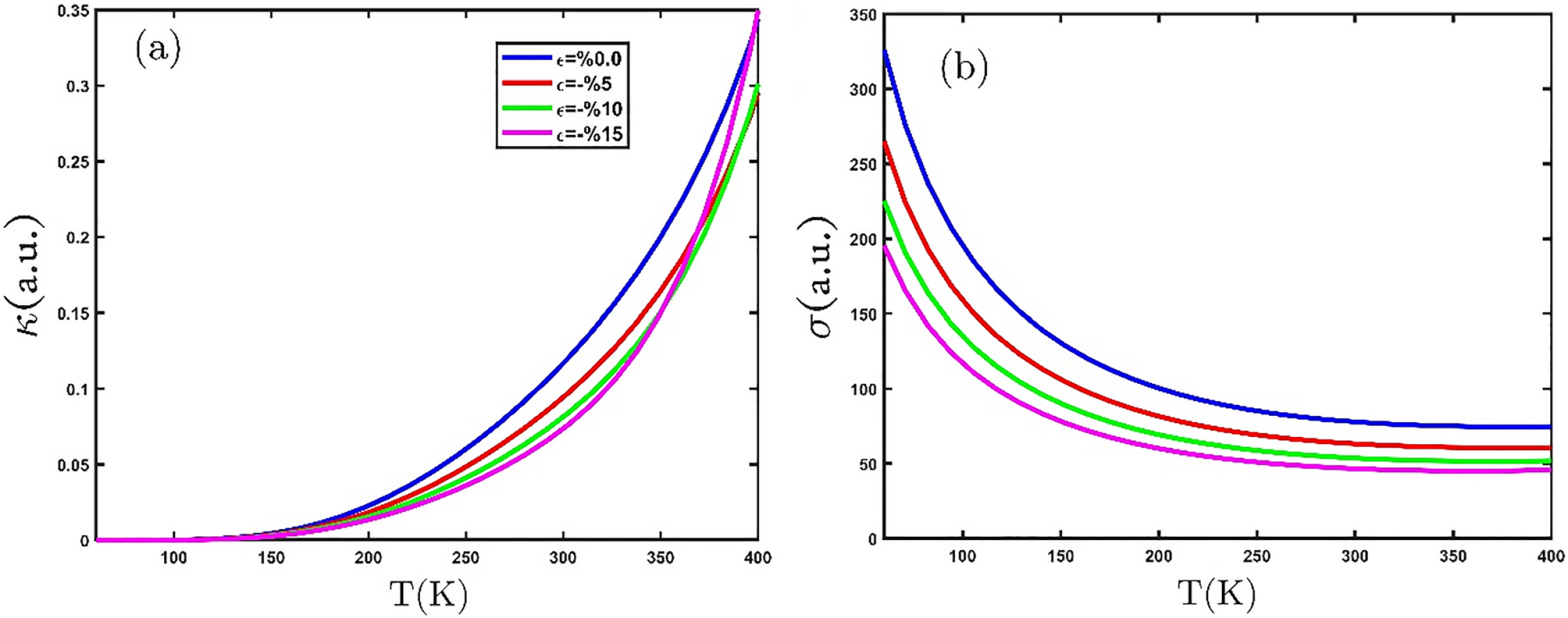
(**a**) The electrical thermal conductivity and (**b**) electrical conductivity as a function of temperature, for several compressive biaxial strains.

Eventually, Fig. 10a and b show the behavior of the electronic thermal conductivity and electrical conductivity with the compressive uniaxial strain for GeCH_3_SL, respectively. In Fig. 10a and b, we observe that applying compressive biaxial strain reduces thermal and electrical conductivity. We find that compressive biaxial strain increases the band gap and prevents more electrons from moving into the conduction band.

**Figure 11:**
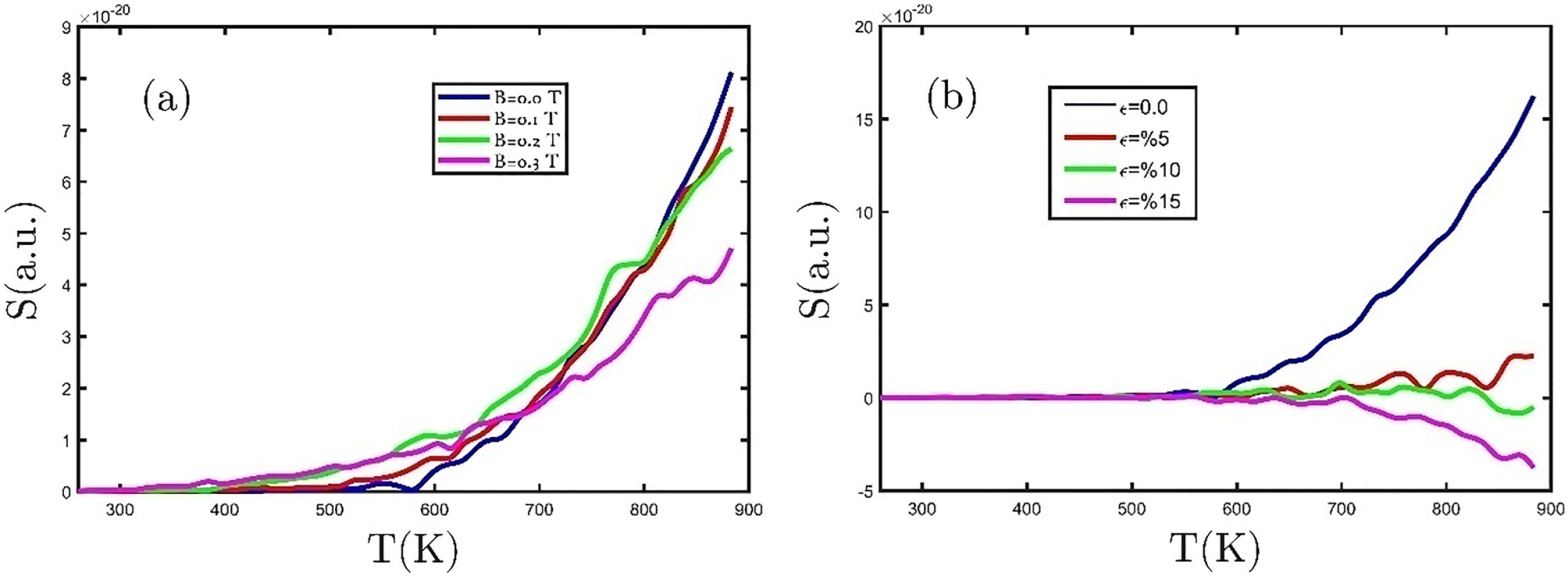
The Seebeck coefficient as a function of temperature (**a**) for different external magnetic field, and (**b**) for several tensile biaxial strains.

Figure 11a exhibits the numerical results of the dependence of the Seebeck coefficient on temperature, which is affected by different external magnetic fields. Furukawa et al. proposed that the Seebeck coefficient sign determines the kind of the majority of the charge carriers^42^. So, the positive (negative) sign of the Seebeck coefficient demonstrates that the charge and heat carriers are transferred by the hole (electron). In Fig. 11a, the sign of the Seebeck coefficient is positive in the whole temperature range, which denotes the GeCH_3_ SL is a p-type semiconductor. Also, by applying an external magnetic field, the sign of the Seebeck coefficient does not change and remains positive. In the following, we want to study dependence of the Seebeck coefficient of the GeCH_3_ SL on the various tensile biaxial strains. The Seebeck coefficient for the GeCH_3_ SL versus temperature for tensile biaxial strains is plotted in Fig. 11b. The Seebeck coefficient sign changes from positive to negative at temperatures above 500 K and at tensile biaxial strain of %15 in Fig. 11b. Therefore, GeCH_3_ SL from a p-type semiconductor to an n-type at tensile biaxial strain of %15.

## Concluding remarks

To summarize, we theoretically examined the thermoelectric, thermodynamic, and magnetic properties of the GeCH_3_SL via the TB model and Green's function approach. The effects of external magnetic field, electron doping, temperature, and tensile and compressive biaxial strain on the electronic thermal conductivity, electrical conductivity, electronic heat capacity, and magnetic susceptibility have been studied. The electronic thermal conductivity and heat capacity increases with increasing temperature, meanwhile magnetic susceptibility, and electrical conductivity decreases. We found that the GeCH_3_SL is a paramagnetic material. Therefore, The GeCH_3_SL alters to antiferromagnetic by electron doping. Furthermore, the electronic heat capacity, magnetic susceptibility, and electronic thermal and electrical conductivity are increased by employing an external magnetic field, electron doping, and tensile biaxial strain. However, compressive biaxial strain yields a decrease in thermoelectric and thermodynamic properties. The sketched properties of the GeCH_3_SL will be very effective for its application in next-generation thermoelectric, spintronic, and valleytronics devices.

## Data Availability

All data generated during this study are included in this published article. In detail, we calculated the thermoelectric and thermodynamic properties of the GeCH_3_ single-layer (SL) by using the tight-binding (TB) and equilibrium Green’s function method. The more calculations detail described in the manuscript, will be freely available to any researcher wishing to use them by connect to the corresponding author.

## References

[1] Rao CNR, Sood AK, Subrahmanyam KS, Govindaraj A (2009). Graphene: The new two-dimensional nanomaterial. Angew. Chemie Int. Ed..

[2] Allen MJ, Tung VC, Kaner RB (2010). Honeycomb carbon: A review of graphene. Chem. Rev..

[3] Novoselov KS (2004). Electric field effect in atomically thin carbon films. Science (80-).

[4] Novoselov KS (2005). Two-dimensional gas of massless Dirac fermions in graphene. Nature.

[5] Geim, A. K. & Novoselov, K. S. The rise of graphene. In *Nanoscience and Technology* 11–19 10.1142/9789814287005_0002.

[6] Tang Q, Zhou Z (2013). Graphene-analogous low-dimensional materials. Prog. Mater. Sci..

[7] Jing Y, Tan X, Zhou Z, Shen P (2014). Tuning electronic and optical properties of MoS2 monolayer via molecular charge transfer. J. Mater. Chem..

[8] Abdi M, Astinchap B, Khoeini F (2022). Electronic and thermodynamic properties of zigzag MoS_2_/MoSe_2_ and MoS_2_/WSe_2_ hybrid nanoribbons: Impacts of electric and exchange fields. Results Phys..

[9] Tao L (2015). Silicene field-effect transistors operating at room temperature. Nat. Nanotechnol..

[10] Azizi F, Rezania H (2021). Spin structure factors of doped monolayer Germanene in the presence of spin-orbit coupling. Sci. Rep..

[11] Jing Y, Tang Q, He P, Zhou Z, Shen P (2015). Small molecules make big differences: molecular doping effects on electronic and optical properties of phosphorene. Nanotechnology.

[12] Rezania H, Abdi M, Astinchap B (2021). Optical absorption of phosphorene structure in the presence of spin–orbit coupling: Mechanical strain effects. Eur. Phys. J. Plus.

[13] Cai Y, Zhang G, Zhang YW (2014). Polarity-reversed robust carrier mobility in monolayer MoS2 nanoribbons. J. Am. Chem. Soc..

[14] Qiao J, Kong X, Hu Z-X, Yang F, Ji W (2014). High-mobility transport anisotropy and linear dichroism in few-layer black phosphorus. Nat. Commun..

[15] Ye X-S, Shao Z-G, Zhao H, Yang L, Wang C-L (2014). Intrinsic carrier mobility of Germanene is larger than graphenes: first-principle calculations. RSC Adv..

[16] Shao Z-G, Ye X-S, Yang L, Wang C-L (2013). First-principles calculation of intrinsic carrier mobility of silicene. J. Appl. Phys..

[17] Liu C-C, Feng W, Yao Y (2011). Quantum spin hall effect in silicene and two-dimensional germanium. Phys. Rev. Lett..

[18] Ni Z (2012). Tunable bandgap in silicene and germanene. Nano Lett..

[19] Bianco E (2013). Stability and exfoliation of germanane: a germanium graphane analogue. ACS Nano.

[20] Ghosh RK, Brahma M, Mahapatra S (2014). Germanane: A low effective mass and high bandgap 2-D channel material for future FETs. IEEE Trans. Electron Devices.

[21] Uematsu Y, Terada T, Sato K, Ishibe T, Nakamura Y (2020). Low thermal conductivity in single crystalline epitaxial germanane films. Appl. Phys. Express.

[22] Coloyan G (2016). Basal-plane thermal conductivity of nanocrystalline and amorphized thin germanane. Appl. Phys. Lett..

[23] Katayama Y, Yamauchi R, Yasutake Y, Fukatsu S, Ueno K (2019). Ambipolar transistor action of germanane electric double layer transistor. Appl. Phys. Lett..

[24] Jiang S (2014). Improving the stability and optical properties of germanane via one-step covalent methyl-termination. Nat. Commun..

[25] Liu Z, Wang Z, Sun Q, Dai Y, Huang B (2019). Methyl-terminated germanane GeCH_3_ synthesized by solvothermal method with improved photocatalytic properties. Appl. Surf. Sci..

[26] Livache C (2019). Optoelectronic properties of methyl-terminated germanane. Appl. Phys. Lett..

[27] Ma Y, Dai Y, Wei W, Huang B, Whangbo M-H (2014). Strain-induced quantum spin Hall effect in methyl-substituted germanane GeCH_3_. Sci. Rep..

[28] Rezaei M, Sisakht ET, Fazileh F, Aslani Z, Peeters FM (2017). Tight-binding model investigation of the biaxial strain induced topological phase transition in GeCH_3_. Phys. Rev. B.

[29] Ma S, Li F, Jiang C (2016). Band-gap modulation of GeCH_3_ nanoribbons under elastic strain: a density functional theory study. J. Electron. Mater..

[30] Papaconstantopoulos DA, Mehl MJ (2003). The Slater Koster tight-binding method: a computationally efficient and accurate approach. J. Phys. Condens. Matter.

[31] Slater JC, Koster GF (1954). Simplified LCAO method for the periodic potential problem. Phys. Rev..

[32] Baroni S, Giannozzi P, Testa A (1987). Green’s-function approach to linear response in solids. Phys. Rev. Lett..

[33] Bir, G. Pikus, G. E., *Symmetry and strain-induced effects in semiconductors*, Hardcover. ISBN 10: 0470073217 ISBN 13: 9780470073216. Publisher: John Wiley & Sons, 1974 ; Publisher.

[34] Mahan GD (2000). Many-Particle Physics.

[35] Kittel C (2004). Introduction to Solid State Physics.

[36] Nolting W, Ramakanth A (2009). Quantum Theory of Magnetism.

[37] Abdi M, Astinchap B (2021). Effect of the magnetic field and electron/hole doping on electronic heat capacity and Pauli spin susceptibility of monolayer MoS2 in the presence of electron-phonon coupling. Mater. Today Commun..

[38] Bruus H, Flensberg K (2004). Many-Body Quantum Theory in Condensed Matter Physics: An Introduction.

[39] Abdi M, Astinchap B (2021). Influence of magnetic field and bias voltage on the thermal conductivity and seebeck coefficient of AA-stacked bilayer SiC. SILICON.

[40] Cullity BD, Graham CD (2011). Introduction to Magnetic Materials.

[41] Chong C, Liu H, Wang S, Yang K (2021). First-principles study on the effect of strain on single-layer molybdenum disulfide. Nanomaterials.

[42] Furukawa S, Ikeda D, Sakai K (2005). Thermomagnetic power and figure of merit for spin-1/2 Heisenberg chain. J. Phys. Soc. Jpn.

